# The Diagnostic Reliability of *BIN1* and *TOMM40* Genotyping in Assessing Dementia Risk

**DOI:** 10.3390/genes16121469

**Published:** 2025-12-08

**Authors:** Marta Machowska, Jerzy Leszek, Aleksandra Mikołajczyk-Tarnawa, Krystyna Głowacka, Elżbieta Trypka, Małgorzata Rąpała, Janusz Piechota, Anna Wiela-Hojeńska

**Affiliations:** 1 Department of Clinical Pharmacology, Wroclaw Medical University, 50-556 Wrocław, Poland; 2 Department of Psychiatry, Wroclaw Medical University, 50-367 Wroclaw, Poland; 3 Screening of Biological Activity Assays and Collection of Biological Material Laboratory, Wroclaw Medical University, 50-556 Wrocław, Poland; 4 Department of Pediatric Surgery, T. Marciniak Lower Silesian Specialist Hospital, 54-049 Wrocław, Poland; 5 Amplicon Ltd., 54-413 Wrocław, Poland

**Keywords:** *BIN1*, *TOMM40*, dementia, Alzheimer’s disease, MCI, biomarkers

## Abstract

Objectives: Alzheimer’s disease (AD) and other dementias represent a growing public health concern, highlighting the need for reliable biomarkers for early diagnosis and treatment monitoring. This study evaluated the potential utility of *BIN1* and *TOMM40* genotyping in diagnosing mild cognitive impairment (MCI) and early-stage dementia. Methods: The *BIN1* rs744373 and *TOMM40* rs2075650 polymorphisms were genotyped in a cohort of 105 individuals diagnosed with MCI or dementia and in 164 cognitively healthy controls. Genotype distributions were compared between the groups, and the potential role of these variants in diagnostic assessment was explored. Results: A significantly higher frequency of the *TOMM40* rs2075650 GG genotype was observed in patients with AD compared with cognitively healthy controls. In contrast, no statistically significant differences in genotype distribution were found among individuals with mild MCI, vascular dementia, or mixed dementia. Furthermore, the distribution of *BIN1* rs744373 alleles did not differ significantly across the analyzed groups. Conclusions: Data on the effects of *BIN1* rs744373 and *TOMM40* rs2075650 polymorphisms in MCI and dementia remain limited and inconsistent. In our study, significant differences were observed only for the *TOMM40* rs2075650 GG genotype and G allele, which were more frequent in Alzheimer’s disease patients than in controls. No significant associations were found for MCI, vascular dementia, or mixed dementia, nor for the *BIN1* rs744373 polymorphism. These results suggest that *TOMM40* rs2075650 genotyping may serve as an additional marker for assessing AD risk.

## 1. Introduction

The etiology of neurodegenerative conditions remains incompletely elucidated. An important role in their pathogenesis is attributed to the interaction of genetic, environmental and lifestyle factors, leading to the development of motor and extra-motor disorders—mainly cognitive and behavioral functions. With the prevalence of dementia expected to double by 2060, attempts are being made to develop a global strategy to deal with this impending crisis. Although there are still no effective treatments for dementia, increased funding for research to understand neurodegeneration offers hope for the future [1]. For early diagnosis and optimization of therapeutic management in patients with neurodegenerative diseases, the search for molecular markers is crucial. A recent comprehensive Mendelian randomization study identified potential biomarkers in cerebrospinal fluid (CSF) and plasma for various types of dementia, including apolipoprotein ε2 (*APOE2)* for various dementias, Siglec-3 for AD in CSF, and CD33 for AD [2]. These findings confirm that genetic biomarkers offer potential for early diagnosis and treatment monitoring, and hold promise as markers of response to immunotherapy [3].

Bridging integrator 1 (BIN1) is a protein which is known to play a crucial role in processing cellular transport in the brain. It affects β-secretase site (BACE1) during initiation of amyloid precursor protein (APP) cleavage and amyloid-β (Aβ) deposition [4]. In microglia, BIN1 regulates proinflammatory and neurodegeneration-related activation, influencing key homeostatic and inflammatory response pathways [5]. Human microglia display substantial heterogeneity, with aging-associated shifts toward senescent and dystrophic phenotypes that diminish their neuroprotective functions. In AD, microglial activation becomes increasingly dysregulated, promoting chronic inflammation and synaptic dysfunction. These findings underscore the need to view microglial states as a continuum and highlight the value of standardized neuropathological scoring in characterizing their role in disease mechanisms and potential therapeutic targets [6]. Studies have shown that *BIN1* expression is altered in AD brains, with a reduction in the largest isoform and an increase in smaller isoforms [7]. It has been shown that this protein accumulates near amyloid deposits in transgenic AD models, suggesting a potential role in extracellular Aβ deposition [8]. The *BIN1* rs744373 polymorphism is associated with accelerated Aβ-related tau accumulation and faster cognitive decline in individuals at risk for AD [9]. Carriers of the risk variant demonstrate reduced working memory performance, enlarged hippocampal volume, and modified functional interactions between the hippocampus and prefrontal regions [10]. Furthermore, this polymorphism is linked to elevated tau pathology, independent of amyloid status and diagnosis, in non-demented elderly individuals [11].

TOMM40 (translocase of the outer mitochondrial membrane 40 homolog), a critical component of the mitochondrial outer membrane translocase complex, plays a key role in protein import and lipid metabolism. It forms the central pore of the TOM complex and actively participates in sorting proteins into different mitochondrial compartments [12]. TOMM40 regulates lipid levels in the liver and plasma lipoproteins via a liver X receptor (LXR)-dependent pathway, influencing triglyceride and cholesterol metabolism [13]. The protein also has a significant impact on neurodegenerative diseases, as it facilitates the import of potentially harmful proteins, includingAPP, Aβ, and alpha-synuclein, all of which can impair mitochondrial function [14]. Additionally, TOMM40 is involved in the PINK1/Parkin-mediated mitophagy pathway, which is crucial for cellular maintenance. Genetic polymorphism has been linked to Alzheimer’s and Parkinson’s diseases in both Caucasian and Asian populations [15]. Preliminary findings from a small prospective study suggested that the presence of the long/long *TOMM40* genotypes correlates with earlier onset of MCI or AD [16]. Investigating the *TOMM40* role in protein import and lipid metabolism may help to better understand age-related neurodegenerative diseases and assess the necessity of therapeutic interventions [13].

Due to ambiguous results from previous studies on the utility of *BIN1* and *TOMM40* genotyping, coupled with the increasing incorporation of molecular biology approaches in identifying biomarkers and therapeutic targets for neurodegenerative disorders, this study aimed to evaluate the potential of polymorphism genotyping in these genes as early markers of neurodegenerative disease onset.

## 2. Materials and Methods

### 2.1. Study Population

**Figure 1:**
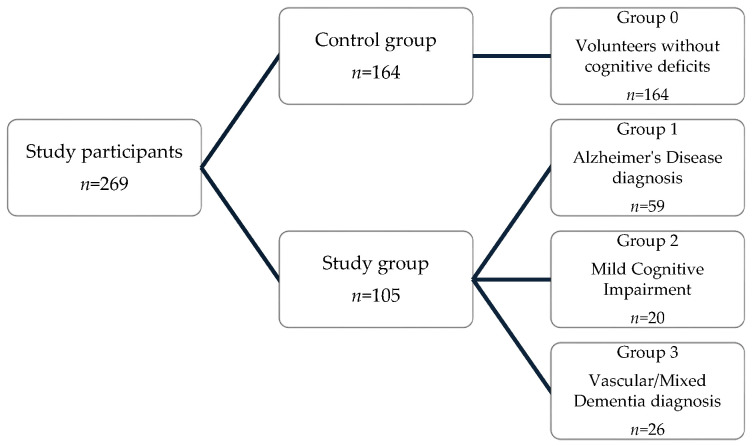
Diagram of study participants.

The study protocol included 269 individuals, comprising cognitively healthy participants (control group) and patients with diagnosed cognitive impairment (study group), which was further divided into several subgroups (Figure 1). The study group consisted of 105 individuals diagnosed with dementia or mild cognitive impairment, including Alzheimer’s disease (*n* = 59), mixed dementia (*n* = 16), vascular dementia (*n* = 10) and mild cognitive impairment (*n* = 20), all hospitalized at the Department of Psychiatry, Wroclaw Medical University. For the purposes of statistical analysis, the study group was divided into subgroups according to the diagnostic criterion: group 1—patients with Alzheimer’s disease, group 2—individuals with mild cognitive impairments, group 3—patients with vascular/mixed dementia. Additionally, genotyping was conducted on 164 age-matched healthy volunteers as a genotype frequency control group (group 0). Dementia was diagnosed in line with Alzheimer’s Association guidelines. Exclusion criteria were: (1) brain injuries, (2) acquired causes of dementia. Patients were informed about the study details and provided written consent for participation in molecular tests and the processing of clinical data without personal identifiers. Questionnaires, completed by an authorized representative, included demographic data (sex, age), anthropometric data (weight), age at diagnosis, duration of dementia, and neuropsychological assessments, including the Mini-Mental State Examination (MMSE) and Montreal Cognitive Assessment (MoCA) results. Characteristics of the study participants are presented in Table 1. The study protocol was approved by the Local Ethics Committee of the Wroclaw Medical University (141/2019).

### 2.2. Genotyping

Peripheral blood samples (2–5 mL) were collected into sterile EDTA-coated tubes, gently inverted several times to ensure adequate anticoagulation and stored at 4 °C until DNA extraction. Prior to isolation, whole blood was equilibrated to room temperature and mixed by gentle inversion to obtain a homogeneous suspension. Genomic DNA was isolated from whole blood using the E.Z.N.A.^®^ Blood DNA Mini Kit (Omega Bio-tek, Norcross, GA, USA) following the manufacturer’s protocol. Briefly, 200 µL of whole blood was lysed in protease-containing buffer, and nucleic acids were selectively bound to a silica membrane. After sequential washing steps, DNA was eluted in 100 µL of nuclease-free water. DNA concentration and purity were assessed using a NanoDrop spectrophotometer (Thermo Fisher Scientific, Waltham, MA, USA), and integrity was verified by electrophoresis on a 1.5% agarose gel. Samples were stored at −20 °C until further analysis. The participants were genotyped for the *BIN1* (rs744373) and the *TOMM40* (rs2075650) using AmpliSNiP Dementia Screening Panel (qPCR) (Amplicon, Wrocław, Poland) on Biometra TOptical 96 system (Analytic Jena, Jena, Germany). Reactions were prepared in a final volume of 20 µL, containing: 12 µL Enzymatic Reagent (including genotyping Master Mix, primers and allele-specific probes labeled with FAM/HEX fluorophores), 10 ng of genomic DNA and water. Thermal cycling conditions were as follows: initial denaturation at 95 °C for 5 min, followed by 35 cycles of denaturation at 95 °C for 10 s and annealing/extension at 58 °C for 25 s. Each reaction was performed in duplicate, and no-template controls were included in each run. Fluorescence data were analyzed using the instrument’s software with automatic allele-calling settings. Genotypes were accepted only when amplification curves showed clear separation of allelic clusters and when duplicate measurements were consistent.

### 2.3. Statistical Analyses

Statistical analyses were conducted using Statistica v.13.3 (TIBCO Software Inc., Palo Alto, CA, USA). Shapiro-Wilk and Levene’s tests were applied to assess normality and homogeneity of variances, respectively. Data are presented as mean ± SD for normally distributed variables or median (Q1–Q3) for non-normal distributions. Categorical variables are expressed as percentages, and intergroup differences were evaluated using the chi-square test (χ^2^; df = (m − 1) × (n − 1)). Continuous variables were compared using the Kruskal-Wallis test, with post hoc two-sided tests for three-group comparisons. A *p* < 0.05 was considered statistically significant.

## 3. Results

A total of 269 individuals participated in this study. They consisted of 164 healthy controls (94 females, 70 males) and 105 patients with cognitive impairments (60 females, 45 males). No significant differences were observed in the distribution of sex or age between the control group and the study group. The average age of patients in Group 2, with MCI, was significantly lower compared to those in Group 1 (Alzheimer’s disease) and Group 3 (vascular and mixed dementia). A similar pattern was observed in the age at which cognitive deficits were diagnosed. Among subjects in the Group 3 (vascular and mixed dementia group), disease duration was the longest and significantly differed from that in Groups 1 and 2. The MMSE scores were notably higher among patients with MCI (Group 2) compared to those with dementia in Groups 1 and 3. In contrast, the highest MoCA scores were observed in patients from Group 3, with significant variations only when compared to the Alzheimer’s disease group (Group 2). No differences were found in the distribution of *BIN1* genotypes between the control group and the study subgroups. However, the distribution of *TOMM40* genotypes differed between the control group and Group 1 (patients with Alzheimer’s disease), with no differences observed between the other study subgroups. A comparative analysis of polymorphism frequencies among the studied groups is shown in Table 2.

## 4. Discussion

Global health strategies are increasingly addressing the rising prevalence of dementia in aging populations worldwide. The World Health Assembly approved a Global Action Plan on Dementia in 2017, focusing on policy, awareness, risk reduction, diagnosis, treatment, care, and research [17].

Recent advances in genomic technologies have significantly expanded our understanding of the genetic profile underlying neurological disorders. Whole-exome and whole-genome sequencing, alongside long-read sequencing, allow detection of both common and rare variants, as well as structural genomic changes, which together contribute to disease susceptibility. These findings underscore the importance of integrating multi-layered data, including genetic, molecular, neuroimaging, and clinical information, to comprehensively characterize disease mechanisms [18].

Particularly noteworthy are also insufficiently acknowledged in the literature personality alterations and behavioral symptoms. Meta-analytic evidence indicates that personality traits remain relatively stable during preclinical stages of dementia and mild cognitive impairment, with minimal observable changes. However, in clinical stages, there is a pronounced increase in neuroticism accompanied by decreases in extraversion and conscientiousness, while changes in openness and agreeableness are more moderate. These findings suggest that significant alterations in personality emerge primarily during advanced disease, highlighting their potential utility as behavioral markers alongside genetic factors for monitoring dementia progression and tailoring patient care [19].

Up-to-date genome-wide association studies have identified additional AD candidate genes, highlighting the complex genetic landscape of the disease. Well-known mutations in *APP*, *PSEN1*, and *PSEN2* genes are primary causes of autosomal dominant early-onset Alzheimer’s disease (EOAD). However, these mutations explain only a small fraction of EOAD cases [20]. Other genetic risk factors include *APOE4* alleles and rare variants in *TREM2, SORL1*, and *ABCA7* genes [21]. Moreover, recent reports have described the role of genetic polymorphisms in *ADAM17/TACE* in the sporadic form of Alzheimer’s disease, as well as their impact on the clinical phenotype of patients. These findings support the role of *ADAM17/TACE* as a potential biomarker for AD risk and cognitive/behavioral profiles, and suggest it may represent a candidate for therapeutic targeting [22]. Overall, the potential of genotyping and assessing the utility of genetic biomarkers has not yet been fully exploited.

Many studies in recent years in various populations have attempted to demonstrate an association between polymorphism in the *BIN1* gene and the risk of Alzheimer’s disease. Significant associations have been found between the single-nucleotide polymorphism (SNP) rs744373 and AD. In the Iranian population, the C allele and CC genotype of rs744373 were more common in AD patients compared to controls [23]. Similarly, a meta-analysis of East Asian populations revealed a significant association between rs744373 and AD risk [24].

The most recent publication on this topic indicates that *BIN1* exerts its influence primarily through regulatory mechanisms, as the majority of its risk variants are located within non-coding intronic or upstream regions, potentially affecting gene transcription, mRNA splicing, and isoform balance. Altered *BIN1* expression has been observed in Alzheimer’s disease brains, with a decrease in neuronal (long) isoforms and an increase in glial (short) isoforms, suggesting cell-type-specific dysregulation. These findings support the role of *BIN1* in tau-related neurodegeneration and highlight its potential as a therapeutic target for modulating disease progression [25].

However, these findings are not consistent with other studies. In the Han Chinese population, while the rs7561528 SNP showed a significant association with AD, no significant difference was observed for rs744373 genotypes between AD patients and controls [26]. Similarly Schaeverbeke et al. found no association between *BIN1* rs744373 and tau-PET load in cognitively intact older adults [27]. In contrast in MCI) populations, Cruz-Sanabria et al. found that *BIN1* rs744373 carriers demonstrated differences in language and memory task performance compared to non-carriers [28].

These conflicting results highlight the complexity of genetic factors in AD and suggest that the association between *BIN1* polymorphisms and AD risk may vary across different populations.

In our study, we did not observe significant differences in genotype distribution between the control group and the patient subgroups with Alzheimer’s disease, mild cognitive impairment, or vascular and mixed dementia. In the parallel statistical analysis none of the *BIN1* rs744373 genotypes were associated with age at diagnosis, disease duration, or scores on cognitive deficit assessment scales.

Interestingly in a Portuguese primary care-based cohort, *BIN1* rs744373 risk-allele carriers were found to have a lower risk of dyslipidemia and respiratory diseases, while tending to have an increased risk of type 2 diabetes [29]. The same study found that *APOE4* carriers had poorer cognitive performance and higher risk of dyslipidemia. Following these observations, there is a possibility that, in the case of *BIN1* polymorphism, investigating correlations between its genotypes and other conditions may allow to find the practical application of this marker.

The *TOMM40* rs2075650 polymorphism has been consistently associated with increased Alzheimer’s disease risk across multiple cohorts. Meta-analyses have shown a significant correlation between this variant and AD susceptibility in both Asian and Caucasian populations [30]. The GG genotype of rs2075650 was linked to a substantially higher AD risk and lower plasma Aβ42 levels in Chinese older adults. Additionally, *TOMM40* interacts with other genes like *PVRL2* to further increase AD risk [31]. Beyond rs2075650, other *TOMM40* variants such as rs157581 and rs11556505 have been associated with AD risk in Taiwanese populations, probably through inducing mitochondrial dysfunction, microglial activation, and neuroinflammation [32]. In a Colombian sample, rs2075650 was significantly associated with late-onset AD, and G allele carriers exhibited an average disease onset 6 years earlier than A allele carriers [33]. This genetic variant interacts with vascular risk factors to influence cognitive performance in dementia-free Chinese older adults, with carriers of the G allele being more vulnerable to cognitive decline when exposed to these risks [34].

Moreover, the *TOMM40* rs2075650 polymorphism has been associated with cognitive decline progression in individuals with mild cognitive impairment. Studies have shown that this polymorphism predicts the two-year outcome of amnestic MCI, with G allele carriers having higher conversion rates to dementia [35]. The polymorphism is also linked to an earlier age of MCI onset and potentially stable MCI phenotype [36]. While some research suggests *TOMM40* SNPs may be associated with progression from MCI to Alzheimer’s disease, these findings require further investigation [37]. A genome-wide association study identified rs2075650 as significantly associated with cognitive aging, although subsequent analyses indicated that this effect might be primarily due to the adjacent *APOE* gene [38]. Conversely another investigation confirmed that the rs2075650-G allele may be an independent risk factor for AD, even in *APOE4* non-carriers [39].

Recent evidence suggests that the *TOMM40* ’523 polymorphism (rs10524523), a variable-length poly-T repeat in intron 6, may influence cognitive trajectories even among individuals with the same *APOE* genotype. In a study of *APOE3* homozygotes, carriers of the “very long” (VL) allele exhibited distinct patterns of improvement in executive function compared to carriers of the short (S) or long (L) alleles [40].

Overall, the *TOMM40* rs2075650 variant appears to play a role in cognitive decline progression, but its exact mechanism and possible connection with *APOE* remain subjects of ongoing research.

In the present study, we confirmed the existing differences in genotype distribution between the control group and the Alzheimer’s disease patient group. The GG genotype and the G allele were significantly more frequent in the patient group, supporting the potential of *TOMM40* rs2075650 genotyping as a biomarker for Alzheimer’s disease onset. No differences in genotype frequency were observed in the remaining patient groups compared to the control group, highlighting the need to investigate the impact of this polymorphism on the occurrence of vascular and mixed dementia. However, it should be noted that functional studies are crucial for determining how specific genetic variants influence molecular pathways involved in neurodegeneration. By assessing their impact on gene expression, protein function, and processes such as neuroinflammation or synaptic integrity, these analyses provide mechanistic insight that complements genetic association findings and supports the development of targeted therapeutic strategies.

### Limitations

A limitation of the study is the small sample size in the MCI, vascular, and mixed dementia groups. This makes it difficult to find statistically significant differences in the distribution of individual genotypes. Increasing the group size could lead to different conclusions. The study was conducted within a relatively homogeneous population, which may limit the generalizability of the findings to more diverse global populations.

## 5. Conclusions

To date, promising research suggests that variants of *TOMM40* or *BIN1* may be relevant for assessing the risk of Alzheimer’s disease; however, they have not yet been recognized as standard diagnostic criteria for the condition. The diagnosis of Alzheimer’s disease primarily depends on clinical symptoms, such as memory impairments and cognitive dysfunction, as well as neuroimaging findings (e.g., magnetic resonance imaging) and biomarkers, such as amyloid-beta and tau levels in cerebrospinal fluid. In our study, we identified an increased frequency of the GG genotype and the G allele among patients with Alzheimer’s disease. These findings may support the inclusion of *TOMM40* rs2075650 genotyping as a clinically relevant diagnostic biomarker in standard diagnostic protocols and potentially accelerate therapy or enhance preventive measures.

## Figures and Tables

**Table 1:** Characteristics of the study participants.

	Control Group	Study Groups					
	Group 0 *n* = 164	Group 1 *n* = 59	Group 2 *n* = 20	Group 1 *n* = 59	Group 3 *n* = 26	Group 2 *n* = 20	Group 3 *n* = 26
Age, y	68.1 (±12.9)	74.5 (±9.6)	56.5 (±15)	74.5 (±9.6)	74.7 (±19)	56.5 (±15)	74.7 (±19)
*p*-Value	*p* = 0.405	*p* < 0.001		*p* > 0.999		*p* < 0.001	
Weight, kg	78.0 (70.0 ÷ 86.5)	74.5 (65 ÷ 83)	77.7 (70 ÷ 82)	74.5 (65 ÷ 83)	80.6 (77 ÷ 87)	77.7 (70 ÷ 82)	80.6 (77 ÷ 87)
*p*-Value	*p* > 0.999	*p* > 0.999		*p* = 0.103		*p* > 0.999	
Age of diagnosis, y	-	67.9 (±9.7)	50.3 (±10)	67.9 (±9.7)	66.3 (±7.1)	50.3 (±10)	66.3 (±7.1)
*p*-Value	-	*p* < 0.001		*p* > 0.999		*p* < 0.001	
Disease duration, y	-	6.60 (±2.26)	6.13 (2.83)	6.60 (±2.26)	8.83 (±1.15)	6.13 (2.83)	8.83 (±1.15)
*p*-Value	-	*p* > 0.999		*p* < 0.001		*p* = 0.003	
MMSE score	28.4 (±1.0)	18.1 (±3.6)	21.6 (±4.9)	18.1 (±3.6)	17.5 (±5.0)	21.6 (±4.9)	17.5 (±5.0)
*p*-Value	*p* < 0.001	*p* = 0.004		*p* > 0.999		*p* = 0.025	
MoCA score	27.9 (±1.3)	19.1 (±3.2)	20.6 (±4.2)	19.1 (±3.2)	21.9 (±2.4)	20.6 (±4.2)	21.9 (±2.4)
*p*-Value	*p* < 0.001	*p* = 0.073		*p* = 0.003		*p* > 0.999	

**Table 2:** Genotype frequency compared between groups.

Gene	rs Code	Participants	Genotype Frequency			χ^2^	*p*-Value
			CT	CC	TT		
*BIN1*	rs744373	Group 0	61 (37.2%)	17 (10.4%)	86 (52.4%)	0.142	*p* = 0.913
		Group 1	23 (39%)	5 (8.5%)	31 (52.5%)		
		Group 0	61 (37.2%)	17 (10.4%)	86 (52.4%)	1.29	*p* = 0.526
		Group 2	6 (30%)	4 (20%)	10 (50%)		
		Group 0	61 (37.2%)	17 (10.4%)	86 (52.4%)	2.43	*p* = 0.297
		Group 3	12 (46.2%	1 (3.8%)	13 (50%)		
			GG	AG	AA		
*TOMM40*	rs2075650	Group 0	8 (4.9%)	53 (32.3%)	103 (62.8%)	5.59	*p =* 0.045
		Group 1	7 (11.8%)	28 (47.5%)	24 (40.7%)		
		Group 0	8 (4.9%)	53 (32.3%)	103 (62.8%)	2.93	*p* = 0.213
		Group 2	0 (0%)	9 (45%)	11 (55%)		
		Group 0	8 (4.9%)	53 (32.3%)	103 (62.8%)	2.15	*p* = 0.342
		Group 3	2 (7.7%)	10 (38.5%)	14 (53.8%)		

## Data Availability

Data access is restricted due to the nature of the study, which involves the comparison of genetic test results. These data are protected under medical confidentiality, the General Data Protection Regulation (GDPR), and Polish national law. Although all participants provided informed consent for genetic testing, the resulting data may only be used for scientific purposes and cannot be shared with third parties. Additional information related to the genotyping methodology or statistical analyses is available from the corresponding author upon reasonable request.

## Abbreviations

The following abbreviations are used in this manuscript:

BIN1	Bridging integrator 1
TOMM40	Translocase of outer mitochondrial membrane 40
MCI	Mild cognitive impairment
AD	Alzheimer disease
CSF	Cerebrospinal fluid
APOE	Apolipoprotein E
CD33	Cluster of Differentiation 33
BACE	Beta-site amyloid precursor protein cleaving enzyme
APP	Amyloid precursor protein
Aβ	Amyloid beta
LOAD	Late-onset Alzheimer’s disease
LXR	Liver X receptor
MMSE	Mini-Mental State Examination
MoCA	Montreal Cognitive Assessment
EDTA	Ethylenediaminetetraacetic acid
qPCR	Quantitative polymerase chain reaction
PSEN	Presenilin
EOAD	Early-onset Alzheimer’s disease
TREM	Triggering receptor expressed on myeloid cells
ABCA	ATP-binding cassette sub-family A
SNP	Single-nucleotide polymorphism
PET	Positron emission tomography
